# Trialing transparent peer review

**DOI:** 10.1186/s13059-017-1314-z

**Published:** 2017-09-12

**Authors:** Andrew Cosgrove, Louisa Flintoft

**Affiliations:** Genome Biology, London, UK

Since our launch in 2000 as one of the first open access journals, *Genome Biology* has always had a strong enthusiasm for open research policies. For example, we were among the first to mandate publicly available supporting data and open source code. Naturally, then, we have watched with close interest as other journals have experimented with—or in some cases wholeheartedly embraced—open or transparent peer review. Among the pioneers in this area were *The BMJ* (which adopted open peer review in 1999 [1]) and the medical journals of the BMC series (which launched with open peer review [2]). Transparent peer review has been championed by journals such as *eLife*, which publishes the editorial decision letter and author responses whenever the author agrees. *Nature Communications* has been piloting transparent peer review since January 2016, with an average 60% of authors voluntarily opting to publish the peer review history of their paper.

Now, to coincide with this year’s Peer Review Week, we are embarking on our own pilot of transparent peer review.

Transparent peer review (in which the reviewers remain anonymous, but the reviewer reports are published alongside the final article if the manuscript is accepted) and open peer review (in which the reviewer reports are published together with the reviewers’ names) increase the accountability of the peer review process; the latter also provides a mechanism for reviewers to obtain credit for their efforts [2]. In addition, when reviewers know that their reports will be published, there is an incentive for them to be more constructive. We have seen an increasing desire among researchers for openness: more and more of our reviewers, particularly within bioinformatics, are choosing to sign their reviews.

Nevertheless, many other researchers feel that the risks associated with totally open peer review still outweigh the benefits. One often-discussed reservation is that early-career researchers will be reluctant to criticize the work of more senior researchers in their field, in case there are negative implications for future grant applications or peer review of their own publications. This could lead to watered-down reviewer reports that hold back on useful criticisms and/or could make it harder to secure reviewers, resulting in extended peer review times. Transparent peer review retains the benefits of open reviewer reports but, because reviewers retain anonymity, some of the drawbacks of open peer review are likely to be diminished. The *Nature Communications* pilot has seen a favorable response from authors, with the majority opting for transparent review [3].

With these pros and cons in mind, we conducted a poll of our editorial board to solicit their views on openness in peer review. A large minority of board members (38%, 13 out of 34 who responded) were in favor of fully open review, with a slightly larger proportion (47%, 16 out of 34) being broadly in favor but with some caveats or equivocacy. The most common suggestion was that anonymous, rather than signed, reviewer reports should be published.

For the *Genome Biology* transparent peer review trial, we will be assessing the degree of support for this approach in our community as well as the impact on peer review times. Half of the manuscripts that are peer reviewed will be assigned to the trial, and the other half will serve as a control arm. For the manuscripts selected for the trial, authors will be offered the option of transparent peer review or standard single-blind review. We are not yet proposing fully open review, but we will give the reviewers the option to reveal their identity.

We will track the time taken to send decisions to the authors of the transparently reviewed manuscripts and will compare these time with those in the control arm of the trial. We will also look at the effect of transparent peer review on the quality of reports, although that is harder to measure. Over the next few weeks, we will be able to establish whether transparent review delays review time, and how much support there is among our author and reviewer communities for the introduction of transparent peer review on a permanent basis.

We will report the results over the coming months. If you have any comments on these proposals, please email us using editorial@genomebiology.com.
